# Challenges of Telemedicine during the COVID-19 pandemic: a systematic review

**DOI:** 10.1186/s12911-022-01952-0

**Published:** 2022-08-03

**Authors:** Racha Ftouni, Baraa AlJardali, Maya Hamdanieh, Louna Ftouni, Nariman Salem

**Affiliations:** 1 Faculty of Medicine, Beirut Arab University, Beirut, Lebanon; 2 Department of Dermatology, American University of Beirut Medical Center, Beirut, Lebanon; 3 Division of Urology, Department of Surgery, American University of Beirut Medical Center, Beirut, Lebanon; 4 Division of Neurosurgery, Department of Surgery, American University of Beirut Medical Center, Beirut, Lebanon

**Keywords:** Challenges, Coronavirus pandemic, COVID-19, Healthcare providers, Patients, Telemedicine

## Abstract

**Background:**

The COVID-19 pandemic has prompted the decrease of in-person visits to reduce the risk of virus transmission. Telemedicine is an efficient communication tool employed between healthcare providers and patients that prevents the risk of exposure to infected persons. However, telemedicine use is not infallible; its users reported multiple issues that complicated the expansion of this technology. So, this systematic review aimed to explore the barriers and challenges of telemedicine use during the pandemic and to propose solutions for improving future use.

**Methods:**

A systematic review was conducted following PRISMA (Preferred Reporting Items for Systematic Reviews and Meta-Analysis) statement. PubMed, Scopus, Web of Science, Academic Search Complete, CINAHL, Embase, and Science Direct were used to look for articles addressing barriers and challenges, in addition to articles proposing solutions. Studies were screened by title and abstract, followed by a full-text review. Risk of bias assessment was done using Critical Appraisal Skills Program for qualitative studies, Newcastle–Ottawa Scale for cross-sectional studies, and A MeaSurement Tool to Assess Systematic Reviews for systematic reviews. After the extraction of data, a narrative synthesis and analysis of the outcomes were performed.

**Results:**

Among 1194 papers identified, only 27 studies were included. Barriers and challenges were assembled under 7 categories: technical aspects, privacy, data confidentiality and reimbursement, physical examination and diagnostics, special populations, training of healthcare providers and patients, doctor-patient relationship, and acceptability. Poor internet connection and lack of universal access to technology were among the technical barriers. Concerns about patient privacy and reimbursement hindered the use of telemedicine too. Physical examination and certain procedures were impossible to perform via telemedicine. Training both healthcare providers and patients was deficient. The doctor-patient relationship was troubled by telemedicine, and both healthcare providers and patients were reluctant to use telemedicine.

**Conclusion:**

Widespread use of telemedicine is still hampered by various barriers and challenges. Healthcare providers should work with various stakeholders to implement the proposed solutions. More research and policy changes are essential to optimize telemedicine utilization.

**Supplementary Information:**

The online version contains supplementary material available at 10.1186/s12911-022-01952-0.

## Background

On the 31st of December 2019, an outbreak of unusual pneumonia cases originated in Wuhan, China. A novel coronavirus was suspected to be the causative organism. Later, the identified virus was named Severe Acute Respiratory Syndrome Coronavirus 2 (SARS-CoV-2) and the disease it causes was known as coronavirus disease of 2019 (COVID-19) [1]. Crossing the boundaries of China, the virus then spread rapidly worldwide. On the 11th of March 2020, the World Health Organization (WHO) announced COVID-19 as a global pandemic [2]. The COVID-19 pandemic is considered the greatest global economic and health challenge of this century [3]. Its effects are still evolving, with more than 185 million cases and 4 million deaths to date [4]. To mitigate SARS-CoV-2 transmission among patients and healthcare workers, the Centers for Disease Control and Prevention (CDC) recommended reducing in-person services. Thus, to slow the spread and reduce the impact of the pandemic, a global shift towards telemedicine arose [5]. Telemedicine, as defined by the WHO, is “healing at a distance”. This means using information and communication technology tools to enhance the quality of care and bypass the barriers imposed by travel [6]. It is a few-decades-old bidirectional technology process involving the interaction of a healthcare provider with a patient, who can access healthcare services from a distance [7]. Telemedicine offers the public an efficient and safe way to consult healthcare professionals about the symptoms of infectious diseases, prevention and treatment measures, psychological troubles, and other issues [8]. Patients can receive medical care remotely without enduring the burden of travel thus decreasing the risk of exposure to highly communicable diseases. This is especially valuable for elderly patients who suffer multiple comorbidities and whose mobility might be limited. From a healthcare provider’s perspective, telemedicine minimizes contact with sick patients, decreasing the transmission of microbes, and preserving the Personal Protective Equipment (PPE) [9]. Telemedicine also reserves an invaluable place in medical education and resident training by maintaining regular learning schedules [10]. Despite the undeniable advantages of telemedicine, its use is still infrequent and relatively unshaped in daily clinical practice [11]. Both the COVID-19 pandemic and the accelerated growth of communication equipment and technology highlighted the importance of telemedicine. Despite the presence of numerous studies appraising the utility of telemedicine as well as the challenges and barriers hindering its optimal implementation, there has been a dearth in studies assessing these challenges in the prevailing pandemic. Subsequently, an update of these challenges was deemed necessary. The rapid spread of cases made the utilization of telemedicine essential to minimize contact and mitigate the transmission of cases as well as cutting down costs and decreasing the time consumed during in-person visits. Therefore, a review of existing literature was established aiming to expose the challenges of telemedicine and to underline recommendations for its future implementation.

## Materials and methods

### Research design and research questions

A qualitative systematic review was conducted. The research questions that were addressed in this review include:

## Research question 1 (RQ1)

What are the challenges and barriers facing patients and healthcare providers utilizing telemedicine services in the COVID-19 era?

## Research question 2 (RQ2)

How to overcome the challenges and barriers facing telemedicine?

The inclusion and exclusion criteria are summarized in Table 1.

**Table 1:** Inclusion and exclusion criteria

Inclusion criteria	Exclusion criteria
1. Articles focusing on the barriers and challenges of using telemedicine during the COVID-19 pandemic or articles discussing both the barriers and their potential solutions	1. Any study that does not answer the research question(s)
2. Articles published in English language	2. Articles published in non-English languages
3. Articles published between December 2019 and 22 August 2020	3. Articles published before December 2019 or after 22 August 2020
4. Qualitative and quantitative observational and interventional studies including systematic and literature reviews	4. Editorials, press/newsletters, commentaries, conference proceedings, case series and case reports and studies that do not provide statistical or theoretical evidence
	5. Full text that cannot be retrieved

### Search Strategy

Guided by the PRISMA statement [12], we conducted our search strategy using seven online databases: PubMed, Scopus, Web of Science, Academic Search Complete, CINAHL, Embase, and ScienceDirect. We used keywords or key search terms combined with Boolean operators (OR/AND) to define our search strategy. The keywords employed in the PubMed search were as follows: ((COVID-19)) OR (COVID19)) OR (coronavirus)) OR (SARS-CoV-2)) OR (NCOV)) AND (telemedicine [MeSH Terms]). MeSH Terms, short for Medical Subject Headings, are controlled vocabulary terms used in PubMed that allow searching for different synonyms of a certain term in the medical literature [13]. Additionally, the keywords ((COVID-19)) OR (COVID19)) OR (coronavirus)) OR (SARS-CoV-2)) OR (NCOV)) AND (telemedicine)) OR (TELEHEALTH)) OR (TELECARE)) OR (E-HEALTH)) OR (MHEALTH)) were used to ensure the consistency of the former search. A Additional file 1 document is available for the search strategy applied in the other databases. The search for relevant articles was conducted between the 21st and 23rd of August 2020 and was restricted to articles published between December 2019 and August 2020. All obtained articles were then imported to EndNote software.

### Study selection

Two reviewers independently screened the studies by title and abstract for the inclusion and exclusion criteria (Table 1). After screening all articles, the two reviewers held online meetings to discuss the included articles. A third reviewer served to solve any disagreements. Consequently, four reviewers conducted a full-text review of the included studies.

### Risk of bias assessment

To assess the risk of bias of the included studies, two reviewers independently assessed each study using a particular assessment tool according to the study design. Critical Appraisal Skill Program (CASP) [14] was used for qualitative studies, which were classified of high quality if the score was 8 or above, of medium quality if the score was 5 to 7, and of low quality, if it was 4 or below [15]. For cross-sectional study designs, the Newcastle–Ottawa Scale (NOS) for cross-sectional studies was employed and studies were classified as very good if the score was 9–10, good if the score was 7–8, satisfactory if the score was 5–6, and unsatisfactory if it was 0–4 [16]. As for systematic reviews, A MeaSurement Tool to Assess Systematic Reviews (AMSTAR) was adopted for evaluation [17].

### Data extraction and synthesis

Two reviewers independently performed the data extraction and synthesis. The extracted data from the included studies can be found in Table 2. Studies were grouped according to the main outcome, i.e., telemedicine challenges and barriers. A narrative synthesis was then conducted, where the extracted data were analyzed, interpreted, relationships deduced, and conclusions drawn out.

**Table 2:** Characteristics of the included studies*

First author /date of publication	Country	Journal	Study design	Telemedicine intervention used	Main findings
Anjana et al. [18] (July 2020)	India	Diabetes Technology and Therapeutics	Cross-sectional	Video, audio, SMS, apps, blogs, TV channels	● Poor telephone connection ● Telemedicine is hard for older patients ● Sensitization and training of providers ● Physical exam may not be appropriate for emergencies ● Not able to check blood pressure ● Telemedicine cannot provide one of the main prerequisites of a successful doctor-patient relationship, namely the human touch ● It will likely be a hybrid method going forward
Anthony Jnr et al. [19] (June 2020)	Norway	Journal of Medical Systems	Systematic review	Telemedicine in general	● Need to provide training to physicians in using telemedicine ● Need to educate patients so that they can be aware of healthcare solutions ● Need to provide laws and upgrade technological infrastructure ● Guidelines to address ethical and legal barriers ● Patient consent ● Physician must notify if any third-party application is being used during a virtual consultation ● Setting of the meeting ● Lack of legislation in developing countries ● The physician must dress professionally, make eye contact with the patient, should try to be friendly and warm, make the patient comfortable ● Verify payment coverage ● Physical exam lacks needed elements of dynamic testing and diagnosis ● Some diagnoses may be difficult to perform virtually ● Preparation for an optimal consultation ● Older patients are least likely to use telemedicine ● Most developing countries may not be able to adopt telemedicine ● Connection problems ● Phone preference over video due to connection ● Funds and support to the healthcare systems to establish telemedicine ●Interstate licensure
Biswas et al. [38] (June 2020)	India	Indian Journal of Palliative Care	Qualitative review	Phone, text messages, smartphone-based applications (WhatsApp, Skype)	● Major limitation of the use of these mobile-based applications is the safety of the patient's data ● Store-forward-delete system ● Lack of multidisciplinary approach over a single call ● Lack of satisfaction among patients
Caetano et al. [39] (June 2020)	Brazil	Cadernos de Saúde Pública	Qualitative review	Telemedicine in general	● Rural populations have difficulties in accessing telemedicine services ● May not be appropriate for certain disorders that impair the patient's ability to use the technology ● Lack of regulation on the use of telemedicine ● Malpractice insurance applied to telemedicine ● Data confidentiality and security ● Establishment of protocols for managing laboratory tests, prescriptions, and scheduling ● No telehealth app can conclusively say whether the patient is infected and require testing in person ● Physical exam and ancillary diagnostic methods cannot be performed remotely
De Simone et al. [20] (June 2020)	Italy	American Journal of Cardiovascular Disease	Qualitative review	Remote monitoring	● Low adherence and cooperation of patients ● Lack of a well-structured organization to manage clinical data ● Some issues concerning the device cannot be managed by remote monitoring ● Need for adequate organization through protocols and guidelines ● Data privacy ●Telemedicine services not uniformly reimbursed across Italy ● Informed consent ● Need for adequate training and updating in the use of systems for all personnel involved ● Not dedicated to the management of emergencies ●Periodic verification of the quality of data and diagnostic tools
Eichberg et al. [21] (July 2020)	USA	Neurosurgery	Systematic review	Telemedicine in general	● Limited access to technology ● Verbal consent ● Providers should have a low threshold to convert to a telephone call ● Telemedicine neurological exam should be considered a screening exam
Ekong et al. [40] (April 2020)	Nigeria	JMIR mHealth and uHealth	Qualitative review	Mobile positioning data	● Balance between deploying technology and maintaining data safety and patient privacy ● Informed consent ● Protect and safeguard individuals' data by law ● A third-party agreement should be formally signed between parties interfacing patient data to protect it
Gao et al. [43] (May 2020)	China	Annals of Translational Medicine	Systematic review and meta-analysis	Telemedicine in general	● People were not followed up for outcomes and hotline data were not collected systematically ● If the operators do not have enough professional knowledge, they may provide wrong information or provide inappropriate medical advice, leading to a treatment delay or missed diagnoses
Jiménez-Rodríguez et al. [22] (July 2020)	Spain	International Journal of Environmental Research and Public Health	Qualitative review	Video consultations	● Lack of access to the required resources and technological difficulties for both professionals and patients (especially for the elderly) ● Some medical procedures are impossible ● Lack of technical skills among professionals and patients ● Need for training regarding both nontechnical and social-emotional skills ● Healthcare professionals were concerned that relationships with their patients may deteriorate ● Problems may arise among patients of advanced age, who may have reduced cognitive abilities
Kalu et al .[23] (August 2020)	UK	Journal of Plastic, Reconstructive, and Aesthetic Surgery	Literature review	Online video consultation platforms and store-and-forward telemedicine	● Time lag and poor audio-visual quality due to insufficient bandwidth ● Transparency over the cost, privacy settings, and relative usage of different systems is limited ● Patient's identity should first be confirmed ● Consent should be gained and recorded ● Ensure that internet connection is secure ● Reassure patients that their privacy is to be respected ● Urgent or serious conditions where physical exam conducted over video consultations may not be appropriate ● It is contraindicated to use video consultations when the provider is unsure of the patient's capacity
Kaplan et al. [24] (July 2020)	USA	International Journal of Medical Informatics	Literature review	Telemedicine in general	● Technological infrastructure ● Access problems (especially the elderly, disabled, or those who have compromised hearing, vision, manual dexterity…) ● Confidentiality, privacy, and security require more scrutiny ● Informed consent ● Ethical concerns ● Regulatory issues ● Doctor-patient relationship ● Patients and clinicians needed to learn how to select and use the technologies ● “a whole-system strategy” is suggested to embed telehealth into routine service and other information system functions
Khilnani et al .[25] (June 2020)	USA	Journal of Information, Communication, and Ethics in Society	Case study	Telemedicine in general	● Older adults and those with economic disadvantage are also more likely to experience digital inequality ● Long-standing challenges that may impact eHealth adoption, including education, income, broadband access, information-seeking skills, and rural residence ● eHealth requires a battery of resources and skills on the part of patient and practitioner ● Older adult patients as more likely to struggle with skill deficits than younger patients ●Digitally disadvantaged are less likely to use eHealth services and thereby bear greater risks during the pandemic to meet ongoing medical care needs during the pandemic
Lawrence et al. [26] (July 2020)	USA	Journal of General Internal Medicine	Case study	Virtual OSCE*	●Technical challenges can result in significant barriers to communication ●Adaptation of traditional components of the medical history and physical exam into the virtual space ● Providers may be unable to acquire basic information from remote patients ● The diagnostic accuracy of the physical exam maneuvers that are self-executed by patients is not yet known ● Residents may not be adequately prepared to provide high-quality care via telemedicine ● Needs for both technical proficiency and care delivery quality assurance at both trainee and practitioner levels ● Many traditionally employed nonverbal cues may be difficult to deploy and/or interpret, both by patients and providers, in a virtual context ● Medical associations recommend at least basic training in technical elements
Moss et al. [27] (July 2020)	USA	Journal of Neuro-Ophthalmology	Cross-sectional	Synchronous (video visits) and asynchronous (Store-forward: remote interpretation of tests, second-opinion review, and e-consults) telehealth	● Data quality was selected as the most perceived barrier ● Video does not offer much more than phone for ophthalmology ● Variable reliability of live video technologies ● Video telemedicine visits may take extra time, resulting in decreased clinic volumes ● Patient dissatisfaction with billing ● Decreased precision and comprehensiveness of examination ● More physically draining than face-to-face to maintain engagement with patients ● Adoption was greatest in the younger respondents ● Provider dissatisfaction ● Privacy ● Protocols, strategies, and scheduling to optimize both efficiency and outcomes and train trainees and providers
Mostafa et al. [28] (July 2020)	Egypt	Journal of Dermatological Treatment	Cross-sectional	Synchronous (video visits) and store-forward	● Lack of teledermatology consultations in the public hospital because of difficult internet connection ● No private insurance coverage for teledermatological services ● Face-to-face visits are still needed for some conditions like skin cancer check and its surgeries ● Showing one part of the body with a skin lesion can be misleading in diagnosis ● Simulated teledermatology visits may miss some diagnoses and complications of medications ● Legislation is needed
Murphy et al. [41] (June 2020)	Ireland	Clinical Orthopedics and Related Research	Systematic Review	Virtual clinic model (video and telephone consultations)	● Administrative error regarding the appointment being issued ● Adverse outcomes encompass complications, further surgeries, deviations from protocols and re-referrals back to the clinic, inappropriate referrals, mismanagement/misdiagnosis, and poorly applied splinting in a specialist hand clinic ● Informed consent and agreement with the treatment plan ● There must be a way for the patient to contact the service if difficulties arise
Ohlstein et al. [29] (August 2020)	USA	The Laryngoscope	Cross-sectional study	Video consultations	● An association between age, technical difficulties, and hesitation in the adoption of virtual medicine ●Increased complaints of logistic and technical difficulties, especially in older populations ● The average age of those declining visits due to technical difficulties was 80 years ● Limitation of virtual otoscopic evaluations ● Lack of physical exam ● Otology patients were less likely to accept a telehealth visit
Puro et al .[30] (June 2020)	USA	The Journal of Rural Health	Cross-sectional study	Telehealth and eICU capabilities	● Internet connectivity ● Technological restrictions ●State reimbursement, regulatory, insurance restrictions play a role in limiting adoptions ● Clinician acceptance barriers, in general, can pose a threat to successful telehealth implementation ● Geographic restrictions ●The concentrations of rural hospitals possessing these capabilities varied widely by state ● Coastal areas lacked to a great extent the capability to provide e-services in rural areas
Rametta et al. [31] (June 2020)	USA	Neurology	Qualitative review	Audio-visual telemedicine encounters and scheduled telephone encounters using phones	● The technical quality was impaired, and the most frequent single causes affecting quality were poor audio, poor video, and interruption of the encounter ● Patients who lacked access to a smartphone or computer application required to enable telemedicine encounters were scheduled for structured (audio-only) telephone encounters ● Access to telemedicine encounters compared to telephone encounters was lower in racial and ethnic minority groups
Serper et al .[32] (August 2020)	USA	Hepatology	Case study	Video consultations	●Technical issues were faced due to software upgrades, resulting in one delayed visit on the same day and one visit requiring rescheduling ● Payer reimbursement policies are highly variable, and most payers do not provide telemedicine parity with in-person visits ● Regulatory and financial barriers
Sorensen et al. [33] (June 2020)	USA	Annals of Surgery	Cross-sectional study	Video consultation and phone calls	● Preference for in-person versus virtual surgical consultation reflected access to care, with a preference for telemedicine decreasing from 72 to 33% when COVID-related social distancing ends ● Telemedicine visits are less appropriate for surgical consultation ●Concerns about technology related to telemedicine: both functionality and data security ● Practical considerations around reimbursement for services and health care utilization will need to be resolved ● Physical exam and establishing trust and comfort could best be done in person ● Initiating/completing a diagnostic workup would also be better in person ● Concern for the depersonalization of care with telemedicine and the ability to establish rapport virtually
Tashkandi et al. [34] (June 2020)	Saudi Arabia	Journal of Medical Internet Research	Qualitative cross-sectional study	Virtual visits	● Lack of physical exam ● Patients’ awareness and access ● IT support and resources were not available ● Lack of physical attendance of the patient ● Lack of a direct doctor-patient encounter ● Medicolegal aspects and privacy ● Only 36.0% will continue virtual management after the pandemic
Tenforde et al. [35] (May 2020)	USA	PM&R: The Journal of Injury, Function and Rehabilitation	Cross-sectional study	Audiovisual consults	● Limitations in technology and ability to perform a physical examination ● Insurance payment models ● Access to telehealth technology ● Physician knowledge ● Malpractice insurance ● Concern regarding the development of patient rapport ● Telehealth visits worked best for follow-up encounters where a more limited physical examination was adequate for management recommendations ● Barriers in healthcare delivery ● Systemic barriers to patients with sensory disabilities, cognitive deficits, those challenged in using technology or without necessary electronic devices, as well as those who require the use of a medical interpreter
Triantafillou et al. [36] (July 2020)	USA	Otolaryngology-Head and Neck Surgery	Qualitative cross-sectional	Video-based consultations	● Technical challenges, including issues with connectivity and audio ● Various aspects of the doctor-patient relationship were studied, including the video aspect, intimacy of telemedicine, the element of ‘‘human touch,’’ and the physical examination ● Anxiety about the logistics of the visit and skepticism regarding telemedicine ● Patients preferred in-person visits and did not think that telemedicine visits could replace in-person ones ● Remote visits hampered the doctor-patient relationship ● The physician could not perform flexible laryngoscopy
Wamsley et al. [42] (July 2020)	USA	Aesthetic Surgery Journal	Case Study	Telephone and Video consultations	● Telehealth utilization is lower among 80 + years individuals this may be due to unfamiliarity and lack of comfort with electronic devices and cognitive decline ● Reasons for the decline of telehealth services included lack of comfort and familiarity with the technology, concerns over privacy and confidentiality, and the preference to schedule an in-person office visit when available ● The legal system currently lags the available technology ● Lack of control over the collection, utilization, and sharing of data over the telehealth systems ● Informed consent ● Malpractice ● The practitioner-patient relationship will inevitably be affected ● The physical nature of many conventional clinical tests is simply impossible to perform
Yoon et al. [37] (June 2020)	USA	International Journal of Spine Surgery,	Qualitative review	Video consultations	● The loss of direct physical examination ● The potential for not detecting subtle neurologic deficits ● Technical software or hardware difficulties ● By no means can telemedicine replace all in-person visits ● There is no standard method to perform a spine examination accurately, reliably, and consistently through telemedicine ● Data privacy is a huge concern ● The breach of personal health information can occur despite multiple layers of security ● These technical shortfalls may be ameliorated by improving network speed, accessibility, and upgrading software usability
Holtz et al. [11] (July 2020)	USA	Telemedicine journal and e-health	Cross-sectional study	Telemedicine in general	● New users of telemedicine perceived more problems hearing the provider through telemedicine more than past users ● Difficulty hearing and seeing the health care provider over the computer/mobile system ● Privacy ● When an unexperienced provider utilizes telemedicine, they might not have the same technical expertise and experience communicating over technology as other telemedicine-only providers ● The health care provider spent little time taking medical history ● Less communication with the provider ● Worries about the accuracy of the information from the telemedicine health care provider ● Worries about the continuity of care

*This table is sorted by alphabetical order of author name

### Data management and registration

The search strategy steps were recorded on a Google Sheet for documentation. All the studies that were scanned for eligibility criteria were imported to EndNote. Each included study was coded by a unique ID. Before study selection, the research protocol was submitted to the PROSPERO register for systematic reviews with the registration number CRD42021242200.

## Results

### Search results

**Fig. 1:**
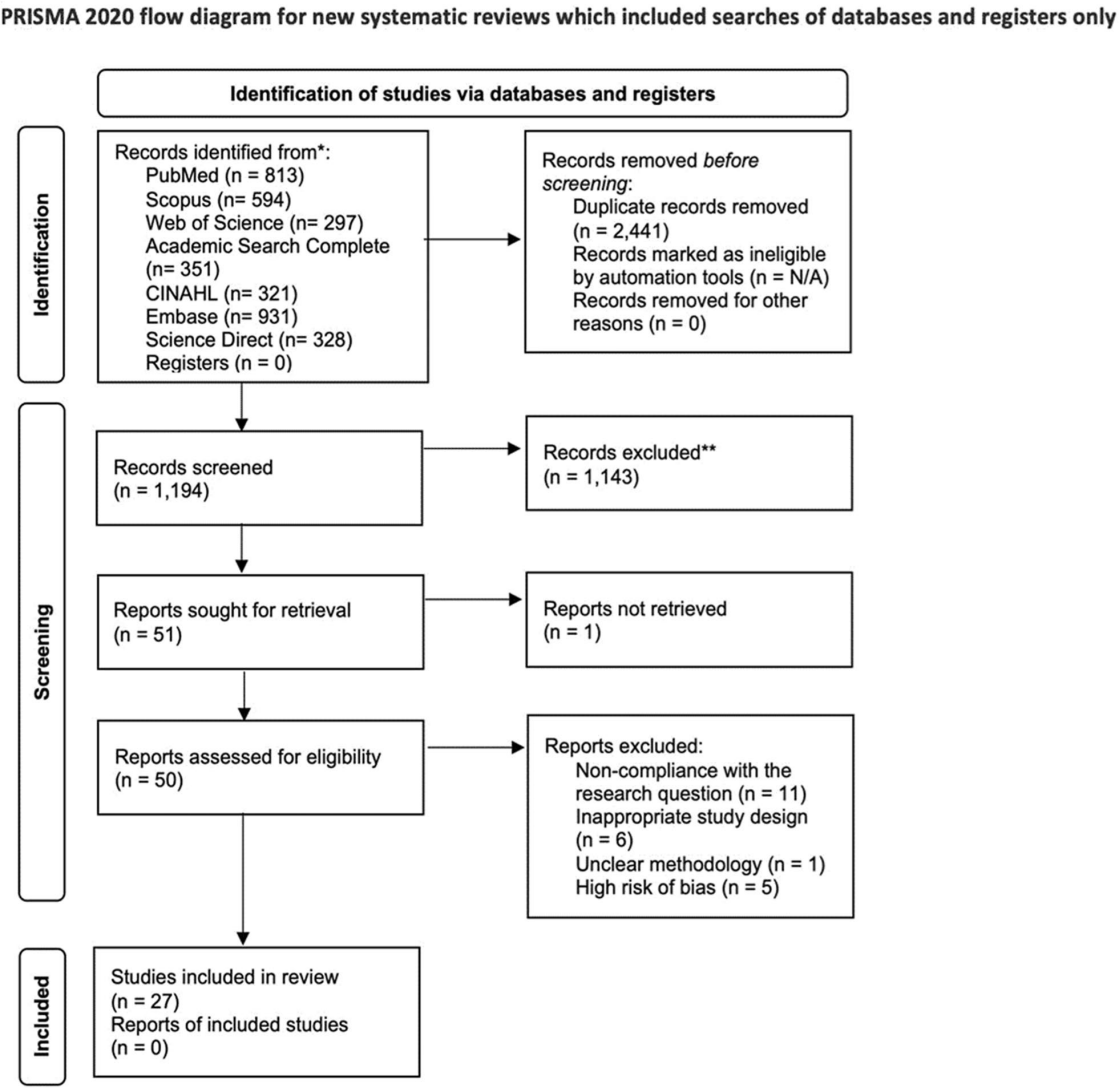
PRISMA flowchart for search strategy

The search result yielded 3635 studies from all seven databases. The number of duplicates found by EndNote’s built-in automatic duplicate function was 2351 and that by manual removal was 90. Hence, the number of remaining papers was 1194. After screening the titles and abstracts for the inclusion and exclusion criteria, 51 papers were left. Twenty four studies were eliminated for the following reasons: 11 did not address the barriers and challenges of telemedicine, 6 had study designs that do not provide statistical or theoretical evidence (2 research letters, 1 case report, 1 case series, 1 brief communication, and 1 quality improvement report), 5 had high risk of bias, 1 paper had no clear methodology, and 1 full text could not be retrieved (Fig. 1)*.* Following full-text review, the final number of studies included reached 27.

### Characteristics of the included studies

The 27 included studies were published in peer-reviewed journals between April 2020 and August 2020. The studies were distributed as follows: 9 qualitative studies, 8 cross-sectional studies, 4 systematic reviews, 4 case studies, and 2 literature reviews. The studies were conducted in 12 different countries: 15 originated from the USA, 2 stemmed from India, and 1 emanated from each of the following: Brazil, China, Egypt, Ireland, Italy, Nigeria, Norway, Saudi Arabia, Spain, and the UK (Table 2).

The main challenges and barriers were grouped under seven themes in order of frequency: technical aspects (n = 21), privacy, data confidentiality and reimbursement (n = 19), physical examination and diagnostics (n = 18), special populations (n = 12), training of healthcare providers and patients (n = 12), doctor-patient relationship (n = 11), acceptability and satisfaction (n = 9). Qualitative studies comprised most of the included studies, with a total of 15 studies assessed through the CASP; 9 of them were of high quality, 5 of medium quality, 1 of low quality. Eight cross-sectional studies were assessed by the NOS, where 4 of them were of good quality and 4 were of very good quality. Four qualitative systematic reviews were included and assessed using AMSTAR; all were of low quality.

#### Technical aspects

Technical issues were the most reported barrier. Twenty-one studies reported on this matter [11, 18–37]. Telemedicine adoption was sluggish and the main obstacles that hindered its rapid implementation were technological prerequisites. The lack of universal access to technology, poor internet connection, and low expansion of rapid internet networks, especially in developing countries were important barriers that impeded communication and interaction through video consultations [18, 19, 21–25, 28, 30, 35–37]. Additionally, the lack of infrastructure and resources constituted a critical challenge [24, 25, 27, 32, 34]. Anthony et al. reported a paucity of high-resolution cameras and high-quality signals [19]. Poor audiovisual quality, latency in the conversation, and time lag also contributed to hampering meaningful communication [11, 22, 23, 27, 31, 36]. Other commonly reported barriers were related to device issues, breakdowns of video consultation platforms, and software upgrades [20, 22, 23, 32, 37]. Some patients also faced difficulties while using or navigating through different telemedicine platforms and needed in-person technical support and information technology (IT) support [23, 34]. Moreover, telemedicine consultations are not uniform across all specialties. For example, in surgical specialties where telemedicine is considered less appropriate, patients might find it difficult to be prepared for the surgery and be examined virtually [33]. Video consultations might not be more helpful than a regular phone call in ophthalmology [27]. Likewise, patients who need a dermatologic consultation may not be able to go through the process of telemedicine due to the absence of teledermatology in public hospitals in some countries like Egypt [28]. All these barriers impelled patients to prefer face-to-face consultations over telemedicine encounters in more than one study [18, 36].

#### Privacy, data confidentiality, and reimbursement

More than half of the included studies [11, 19–21, 23, 24, 27, 28, 30, 32–35, 37–42] tackled the issues of privacy and data security, reporting them as a major barrier for telemedicine visits [27, 34, 38]. Patients feared telemedicine visits due to concerns regarding privacy and confidentiality [11, 19, 21, 24, 33, 34, 37, 40, 42]. These concerns remain a major limitation because it is crucial to utilize technology in healthcare delivery without infringing patient data [40]. Several issues were pointed out to ensure the preservation of patient’s privacy. Eight studies emphasized obtaining informed consent [19–21, 23, 24, 40–42], which should follow the countries’ legislation and should not differ from face-to-face consultations [19, 42]. Adequate information must be provided to patients and clarity should be assured followed by documentation [41]. Moreover, the provider should notify the patient about the use of any third-party application during a telemedicine consultation because of the accompanying cybersecurity risk, and the possibility of breaching the patient's data while using these applications [19, 42]. Wamsley et al. stated that “smartphone apps tracking medical history and personal health measures have been found to share information with third parties” [42]. Consequently, formal agreements with third parties should be made to ensure the preservation of patient data security [40]. Malpractice and liability were also among the barriers [23, 24, 27, 35, 39, 42]. One paper stated that claims could be raised against a provider for a telemedicine consultation just like face-to-face visits [42]. However, in online consultations, the data obtained from the patient is restricted which could place the patient and the provider at risk [23]. During the pandemic, the US federal acts shielded healthcare providers from liability of providing services through telemedicine platforms [27]. Further, reimbursement was a major hurdle in the way of delivery of telemedicine not only being inadequate but absent sometimes [19–21, 27, 28, 30, 32, 39]. With the emergence of the pandemic, Centers for Medicare and Medicaid Services (CMS) and insurers increased their coverage to the level of in-person visits [27]. Despite this advantage, concerns were raised among providers and patients [11, 34, 35]. First, future reimbursement is uncertain [27]; in a cross-sectional study, all physicians reported that they would carry on providing consultations via telemedicine if reimbursement continues [35]. Second, virtual visits were not reimbursed in some countries like Norway, Brazil, Italy, and Egypt [19, 20, 28, 39]. Third, legislation and regulations for telemedicine visits reimbursement are lacking [39]. Another perceived barrier was physicians’ inability to practice out-of-state according to the USA interstate licensure [19, 32, 42].

#### Physical examination and diagnostics

As reported in 18 studies, physical examination and diagnosis-related concerns were among the major challenges in telemedicine visits [18–23, 26–29, 33–37, 39, 41, 42]. Physical examination is arduous to be performed remotely [19, 20, 22, 29, 34–37, 39, 42] because some of its essential elements such as monitoring the vital signs (e.g., measuring the blood pressure) could not be achieved virtually and if to be done are inaccurate [18, 19, 26]. Some medical procedures and diagnostic tests are also impossible at distance [42, 43]: telemedicine visits were deemed to be inadequate especially for surgical specialties [33]. Flexible laryngoscopy, otoscopic evaluation, and ophthalmoscope-based virtual visits are inappropriate [27, 29, 36]. A qualitative paper noted the lack of consensus on one standard procedure for virtual spine examination [37]. Eichberg et al. showed that telemedicine-based neurological examination is of lower quality than that done in-person [21]. Besides, no technology exists that allows palpation at a distance [42]. Patients considered that physical examination and ancillary diagnostic tests are most precise, accurate, and thorough when done in-person [27, 33] and thus were more likely to reject telemedicine visits [29]. This matches the findings of Eichberg et al. [21] who showed that 18.5% of unsuccessful visits were because patients require further assessment and the findings of Murphy et al. [41] who noted that wrong referrals and poor diagnosis and management were more likely to result from a telemedicine visit. Also, many conditions still require in-person evaluation [28] and physical examination may be particularly impractical for emergency conditions [18]. When compared to video consultations, telephone interviews were restricted to verbal communication and descriptions only [42].

#### Special populations

Challenges faced by special populations using telemedicine during the COVID-19 pandemic were discussed in twelve studies [18, 19, 22–25, 29–31, 35, 39, 42]. Age, technological challenges, and reluctance to utilizing telemedicine services are closely correlated [29]. The mean age of patients who denied telemedicine consultations due to technical difficulties was around 80 years [29, 42]: they find it difficult to acquire the digital literacy needed and they have insufficient access to technological advances (e.g., laptop, smartphone, …) [22, 25]. The elderly prefer in-person visits, as they are more reliable and easier to conduct [18], and are more reluctant to use telemedicine and its interventions [19]. Further, they are more likely to require assistance in using telemedicine services, particularly if they have diminished cognitive functions [22]. Demographic disparities have also been implied as an obstacle to telemedicine implementation. People living in rural areas struggle more to access health services and specialists [39], they also suffer a shortage of the internet due to technical reasons [25]. Other vulnerable populations also tend to be digitally disadvantaged: patients belonging to lower socioeconomic class [25], care home residents [23], patients living with certain disabilities (e.g., vision and hearing problems), patients with limited mobility, and non-English writers and speakers whose encounter requires the assistance of a medical interpreter [24, 35]. Patients from ethnic and racial minorities were more likely to perform virtual encounters through telephone rather than through video-based platforms [31].

#### Training of healthcare providers and patients

Twelve papers brought up the lack of training of healthcare providers and patients [11, 18–20, 22, 24–26, 35, 39, 41, 43]. Deficits in technical skills and suitability were noted among both parties [22, 25, 35]. Jimenez et al. [22] pointed out the difference in interactions via telemedicine and the non-technical, social, and economical skills that may not be well handled in a virtual encounter. Several studies concluded that training healthcare providers and patients for using telemedicine technologies is needed [18–20, 22, 24, 26]. Key needs for healthcare providers identified in one study were technical proficiency, proper virtual history taking, virtual physical examination skills, and interpersonal communication skills, yet training is still scarce [26]. Additional barriers to telemedicine delivery were identified including connecting and initiating a video visit [22, 31], non-systematic collection of data, and inability to follow up patients [43]. Staff management, electronic medical record integration, and platforms for documentation and orders were also among the challenges. One study stressed the insufficient knowledge of healthcare providers as a cause of misdiagnosis and delay in management [43].

#### Doctor-patient relationship

Challenges related to doctor-patient relationship were underscored in eleven studies [11, 18, 19, 22, 24, 26, 33–36, 42]. Virtual visits impeded the doctor-patient relationship and many patients opted for in-person visits [36, 42]. Several studies reported that patients and providers had concerns regarding the establishment of this rapport and its continuity as patients may believe that video consultations distance them from their healthcare provider and might create a relationship tainted by mistrust [22, 35]. A cross-sectional study showed that most respondents find comfort and trust when the visits are done in-person, and some insist on seeing their doctor before the surgery [33]. Telemedicine visits lack an essential element of the doctor-patient relationship that is the human touch [18, 36, 42]. The lack of patient’s physical presence and psychological support were also reported. Patients may not be capable of conveying all their concerns compared to in-person visits, and patients said that they feel relieved when they see their doctor in the office [11, 18, 34]. Non-verbal communication and cues “such as allowing for silence, open posturing, and empathetic touch” which could help discern patients’ worries are unfeasible through the virtual platforms [26, 36]. Claims around depersonalization and the absence of intimacy emerged with telemedicine use [20, 24, 27, 29, 30, 33, 34, 36, 38, 42].

#### Acceptability and satisfaction

Nine papers highlighted the issues of acceptability and satisfaction [20, 27, 29, 30, 33, 34, 36, 38, 42]. Healthcare providers and patients refusing the concept of telemedicine encounters [22, 27, 30] and uncertainty and apprehension about telemedicine visits were also reported as a major barrier especially when they are unfamiliar with the technology [36, 42]. An Indian study showed that 34% of patients using telemedicine services were generally less satisfied [38]. Acceptance was variable between specialties; in Ohlstein et al. study [29], the acceptance rate was higher among plastic surgery patients compared to otolaryngology patients. In the De Simone et al. study, the patients’ compliance with telecardiology programs was low [20]. Patients and healthcare providers favored in-person visits over telemedicine encounters and only 33% of patients and 36% of physicians would continue using telemedicine when the pandemic is over [33, 34].

## Discussion

Before the COVID-19 pandemic, telemedicine adoption was low and the idea of undergoing health visits via a virtual platform was not plausible for neither healthcare providers nor patients [44]. The swift upsurge in COVID-19 cases and worldwide lockdowns urged the use of telemedicine as an alternative to in-person visits [8]. However, the prodigious shift towards telemedicine use revealed many shortcomings to a supposedly ideal resort during times of total lockdown. The primary objective of this review was aimed at addressing the challenges and barriers in the way of successfully implementing telemedicine. The secondary objective was to propose solutions and provide recommendations that could improve telemedicine usage during the COVID-19 pandemic and beyond. Although the included studies mainly focused on barriers, many solutions were suggested.

In comparison to other systematic reviews assessing telemedicine, this review reported similar barriers to existing and previous literature. For example Khoushranejad et al. [45] shared the same challenges and barriers of this study. However, these barriers varied in order; While technical aspects were the most reported barrier in our review, acceptance of technology was the most cited challenge in the aforementioned study. In another study the slow internet speed—which falls under the technical aspects- was the most cited barrier followed by skepticism and lack of acceptance in addition to lack of laws and regulations [46]. In contrast, acceptability and satisfaction was the least reported barrier in our review with only 9 citations [20, 27, 29, 30, 33, 34, 36, 38, 42]. This dispersion in reports of barriers might be explained by a cause-effect relationship between these barriers. For example a slow internet connection or inadequate training might be an influencing factor behind refusing utilization of telehealth services in lieu of in-person visits.

With regards to technical difficulties faced during practice, developing countries and rural areas lacked the internet speed needed for synchronous videoconferencing [6]. This necessitates the support of governments by increasing the bandwidth of internet networks and the installment of higher generations of network technologies. Poor infrastructure was a significant hassle in developing countries, which calls for national efforts to provide the adequate strategy, planning, and provision of resources to maintain a solid groundwork for delivery of virtual consultations without interruptions or delays [6]. Before each online consultation, the patient and the provider should be advised to check the functioning of the camera, the microphone, and the internet connectivity to prevent any latency in communication. An IT technician is indispensable should any issue arise. Choosing a standard platform for all consultations, preferably ones that patients are familiar with and are comfortable navigating through could help surmount the process of downloading and registering on a new platform. This approach has previously proven efficient [47], where a pre-made handout containing a set of guidelines about setting and preparing for a telemedicine visit can be used to ease the difficulties faced by patients using the technology.

While developing countries struggle the most with infrastructure and resources, developed countries face more difficulties with legal issues like patient privacy [6]. No legal framework exists to guide the use and advocate for the expansion of telemedicine [39, 48]. In a study from Brazil, physicians said they wanted regulations to provide teleconsultations [39]. Regarding informed consent, it is recommended to educate the patient about the risks and benefits of teleconsultation before starting. In the USA, due to the high demand for telemedicine, the Secretary of Health and Human Services issued a letter protecting all healthcare providers from medical liability [49]. Outside the COVID-19 pandemic, the medical liability outline is unclear. Setting well-formed legislation would permit all parties, namely healthcare providers, patients, and platforms, to recognize their responsibilities and protect them from exploitation. Regarding reimbursement, the CMS and insurance companies in the USA cover telemedicine visits, an approach similar to that followed in Germany [50]. However, this is not the case universally [19, 20, 28, 39]. Nationwide legislations are necessary to cover the expenses of telemedicine should its use be widespread. Whilst physical examination consisted mainly of inspection in some specialties like dermatology, it was impractical when the physician needed to use an ophthalmoscope or laryngoscope. Therefore, technology should be adapted to compensate for the loss of physical factors and instructions should be set to discern whether an in-person visit cannot be deferred, preventing inappropriate diagnoses, referrals, and waste of resources.

Despite the added contribution to physical findings by patient-assisted maneuvers [51], this field is still understudied for practicality and effectiveness taking into consideration that it is governed by several extraneous factors like patient’s literacy and abilities, quality of images and videos [19], and the provider’s expertise. Examination through a virtual platform is not appropriate in cases of emergency and high-risk conditions [21, 23]. Rather, it is advised that telemedicine can be used as a screening tool to triage patients or for follow-up [21, 28, 37]. A comprehensive physical examination is essential for telemedicine to be reimbursed after the pandemic [51]. Many older patients are hesitant to use telemedicine services because they are unfamiliar and lack the technical skills to undertake a virtual consultation. In addition, some adults do not have access to the internet or even telecommunication devices. Consequently, education is demanded for seniors to guide them about the use of the technology and its benefit to them. User-friendly applications would also lessen the difficulties of usage [6]. People living with disabilities should always have assistance to receive a quality of care that is level with the rest of the population. Racial and ethnic minorities should also be taken into account as they have a greater need for healthcare and have less access to telemedicine [52]. Training both healthcare providers and patients is paramount to an efficacious telemedicine encounter. It aims towards increasing their skill set, enhancing their abilities, and boosting their confidence during virtual encounters [6]. Such training may be given by providers accustomed to using the technology who can arrange training for the inexperienced ones. In academic programs, the incorporation of training in well-structured curriculums would increase the readiness of reluctant providers to adopt telemedicine services [26].

Concerning the doctor-patient relationship, an attitude of professionalism should be affirmed while maintaining eye contact and ensuring a welcoming environment for the patients, who will feel more comfortable expressing their concerns. Both physicians and patients must keep interruptions from the environment at a minimum. They should also check the appropriateness of the setting of the meeting, such as sitting in a well-lit room, adjusting the camera position, and for patients, wearing comfortable clothing would ensure a smoother examination [53, 54]. Yet, an essential component of the visits is still lacking virtually, namely the body language which helps in deciphering patients’ reactions when learning about their diagnosis, plan of management, or prognosis [41, 42]. The gap in understanding patients’ concerns and feelings might lead patients and healthcare providers to refrain from using telemedicine. Lastly, some patients are unaware of telemedicine as an alternative for real-time visits which creates an additional barrier [55].

Concerning satisfaction of patients and healthcare providers with telemedicine, multiple studies showed no difference in overall satisfaction between virtual and in-person visits [56, 57].We believe that patients and healthcare providers' reluctance towards using telemedicine stems from the barriers revolving around it. Tackling each barrier at a time would ease the hesitancy and increase the likelihood of accepting and adopting telemedicine over time. Further challenges that should not be missed namely environmental factors including the effects of telemedicine on climate change where telemedicine participated in decreasing carbon emissions by reducing transport emissions [58, 59]. However, other critics argue that the electronic waste generated by telemedicine might pose health hazards as well as environmental pollution [60].

### Strengths and limitations

This review has various strengths and limitations. There is little research particularly systematic reviews examining telemedicine in COVID-19. Despite the presence of other systematic reviews, this study is the most comprehensive. In addition, to ensure an exhaustive literature review, seven databases were used.

Regarding limitations, studies published in languages other than English were excluded. Moreover, grey literature and unpublished papers were not searched which might have led to missing some relevant studies. Furthermore, included studies were mostly qualitative which lack more objective quantitative evidence and the fact that the literature is expanding at a rapid rate makes the evidence evolving and changing over time.

## Conclusion

Telemedicine is a relatively innovative technology employed during the pandemic. Barriers to its widespread use exist and were more pronounced during the COVID-19 pandemic, including technical aspects, privacy, data confidentiality and reimbursement, physical examination and diagnostics, special populations challenges, training of healthcare providers and patients, doctor-patient relationship, and acceptability. Various stakeholders should implement proposed solutions to overcome the difficulties during health crises and beyond.

## Supplementary Information

**Additional file 1.** Supplement 1.

## Data Availability

The datasets used and/or analysed during the current study are available from the corresponding author on reasonable request.

## Abbreviations

AMSTAR: A measurement tool to assess systematic reviews
CASP: Critical appraisal skills program
CDC: Center for disease control and prevention
CMS: Centers for medicare and medicaid services
COVID-19: Coronavirus disease of 2019
IT: Information technology
NOS: Newcastle–Ottawa scale
PPE: Personal protective equipment
PRISMA: Preferred reporting items for systematic reviews and meta-analysis
SARS-CoV-2: Severe acute respiratory syndrome coronavirus 2
WHO: World Health Organization
