# Supplementary material for: Challenges of Telemedicine during the COVID-19 pandemic: a systematic review
Source: BMC Med Inform Decis Mak. 2022 Aug 3;22:207. doi: 10.1186/s12911-022-01952-0 (PMC9351100; doi:10.1186/s12911-022-01952-0)
Supplement: Supplementary file 1 — Additional file 1. Supplement 1. [file 12911_2022_1952_MOESM1_ESM.docx]

Search strategy:

| Database | Keywords | Result (n) | Additional comments |
| --- | --- | --- | --- |
| PubMed | **((COVID-19)) OR (COVID19)) OR (coronavirus)) OR (SARS-CoV-2)) OR (NCOV)) AND (telemedicine[MeSH Terms])** | 813 | MeSH terms search yielded same results |
| Scopus | **"COVID-19" OR "NCOV" OR "CORONAVIRUS" OR "SARS-COV-2" AND "TELEMEDICINE" OR "TELEHEALTH" OR "TELECARE" OR "E-HEALTH" OR "MHEALTH"** | 594 |  |
| Web of Science | **AB=((COVID-19 OR COVID19 OR NCOV OR CORONAVIRUS OR SARS-COV-2)**  **AND**  **(TELEMEDICINE OR TELEHEALTH OR TELECARE OR E-HEALTH OR EHEALTH OR MHEALTH)**  **))** | 297 |  |
| Academic Search Complete | **TELEMEDICINE OR TELEHEALTH OR TELECARE OR E-HEALTH OR EHEALTH OR MHEALTH AND TELEMEDICINE OR TELEHEALTH OR TELECARE OR E-HEALTH OR EHEALTH OR MHEALTH** | 351 |  |
| CINAHL | **COVID-19 OR COVID19 OR NCOV OR CORONAVIRUS OR SARS-COV-2**  **AND**  **TELEMEDICINE OR TELEHEALTH OR TELECARE OR E-HEALTH OR EHEALTH OR MHEALTH** | 321 |  |
| Embase | **COVID-19 OR COVID19 OR NCOV OR CORONAVIRUS OR SARS-COV-2**  **AND**  **TELEMEDICINE OR TELEHEALTH OR TELECARE OR E-HEALTH OR EHEALTH OR MHEALTH** | 931 |  |
| ScienceDirect | **("COVID-19" OR "NCOV" OR "CORONAVIRUS" OR "SARS-COV-2") AND ("TELEMEDICINE" OR "TELEHEALTH" OR "TELECARE" OR "E-HEALTH" OR "MHEALTH")** | 328 |  |

Newcastle-Ottawa Scale

| Study ID | Name | Selection | Comparability | Outcome |
| --- | --- | --- | --- | --- |
| 1 | Anjana et al | ★★★★ | ★★ | ★★★ |
| 17 | Moss et al | ★★★★ | ★★ | ★★ |
| 18 | Mostafa et al | ★★★★ | ★★ | ★★ |
| 21 | Ohlstein et al | ★★★★ | ★★ | ★★ |
| 23 | Puro et al | ★★★★ | ★★ | ★★ |
| 26 | Sorensen et al | ★★★★ | ★★ | ★★ |
| 28 | Tenforde et al | ★★★★ | ★★ | ★★ |
| 32 | Holtz et al | ★★★★ | ★ | ★★ |

CASP

| Study ID | Name | Was there a clear  statement of the aims of  the research? | Is a qualitative  methodology  appropriate? | Was the research  design appropriate to  address the aims of the  research? | Was the recruitment  strategy appropriate to  the aims of the  research? | Was the data collected in  a way that addressed the  research issue? | Has the relationship  between researcher and  participants been  adequately considered | Have ethical issues been  taken into consideration | Was the data analysis  sufficiently rigorous | Is there a clear statement  of findings | How valuable is the  research? |
| --- | --- | --- | --- | --- | --- | --- | --- | --- | --- | --- | --- |
| 3 | Biswas et al | Y | Y | Y | N | Y | T | N | Y | Y | Y |
| 5 | Caetano et al | Y | Y | Y | Y | Y | T | T | Y | Y | Y |
| 6 | De Simoneet al | Y | N | T | N | T | T | T | Y | Y | Y |
| 8 | Ekong et al | Y | Y | Y | T | T | T | T | Y | Y | Y |
| 11 | Jiménez et al | Y | Y | Y | Y | Y | Y | Y | Y | Y | Y |
| 12 | Kalu et al | Y | Y | Y | Y | Y | T | T | Y | N | Y |
| 13 | Kaplan et al | Y | Y | Y | Y | Y | T | T | Y | Y | Y |
| 14 | Khilnani et al | Y | N | Y | T | T | T | T | Y | Y | Y |
| 16 | Lawrence et al | Y | Y | Y | Y | Y | T | T | N | Y | Y |
| 24 | Rametta et al | Y | Y | Y | Y | Y | T | T | Y | Y | Y |
| 25 | Serper et al | Y | Y | Y | Y | Y | N | N | Y | Y | Y |
| 27 | Tashkandiet al | Y | Y | Y | Y | Y | N | Y | Y | Y | Y |
| 29 | Triantafillouet al | Y | Y | Y | Y | Y | Y | Y | Y | Y | Y |
| 30 | Wamsley et al | Y | Y | Y | N | Y | N | Y | Y | Y | Y |
| 31 | Yoon et al | Y | N | N | Y | Y | Y | Y | Y | Y | Y |

Yes :Y

No :N

Can’t tell:T

| **First author / Date of publication** | **Country** | **Journal** | **Study design** | **Telemedicine intervention used** | **Main findings** |
| --- | --- | --- | --- | --- | --- |
| Anjana et al.  July 2020 | India | Diabetes Technology and Therapeutics | Cross-sectional | Video, audio, SMS, apps, blogs, TV channels | - Poor telephone connection - Telemedicine is hard for older patients - Sensitization and training of providers - Physical exam may not be appropriate for emergencies - Not able to check blood pressure - Telemedicine cannot provide one of the main prerequisites of a successful doctor-patient relationship, namely the human touch - It will likely be a hybrid method going forward |
| Anthony Jnr et al.  15 June 2020 | Norway | Journal of Medical Systems | Systematic review | Telemedicine in general | - Need to provide training to physicians in using telemedicine - Need to educate patients so that they can be aware of healthcare solutions - Need to provide laws and upgrade technological infrastructure - Guidelines to address ethical and legal barriers - Patient consent - Physician must notify if any third-party application is being used during virtual consultation - Setting of the meeting - Lack of legislation in developing countries - The physician must dress professionally, make eye contact with the patient, should try to be friendly and warm, make the patient comfortable - Verify payment coverage - Physical exam lacks needed elements of dynamic testing and diagnosis - Some diagnoses may be difficult to perform virtually - Preparation for an optimal consultation - Older patients are least likely to use telemedicine - Most developing countries may not be able to adopt telemedicine - Connection problems - Phone preference over video due to connection. - Funds and support to the healthcare systems to establish telemedicine - Interstate licensure |
| Biswas et al.  June 2020 | India | Indian Journal of Palliative Care | Qualitative review | Phone, text messages, smartphone-based applications (Whatsapp, Skype) | - Major limitations of the use of these mobile-based applications is the safety of the patient's data - Store-forward-delete system - Lack of multidisciplinary approach over a single call - Lack of satisfaction among patients |
| Caetano et al.  June 2020 | Brazil | Cadernos de Saúde Pública | Qualitative review | Telemedicine in general | - Rural populations have difficulties in accessing telemedicine services - May not be appropriate for certain disorders that impair the patient's ability to use the technology - Lack of regulation on the use of telemedicine - malpractice insurance applied to telemedicine - Data confidentiality and security - Establishment of protocols for managing laboratory tests, prescription and scheduling. - No telehealth app can conclusively say whether the patient is infected and require testing in person - Physical exam and ancillary diagnostic methods cannot be performed remotely |
| De Simone et al.  June 2020 | Italy | American Journal of Cardiovascular Disease | Qualitative review | Remote monitoring | - Low adherence and cooperation of patients - Lack of a well-structured organization to manage clinical data - Some issues concerning the device cannot be managed by remote monitoring - Need for adequate organization through protocols and guidelines - Data privacy - Telemedicine services not uniformly reimbursed across Italy - Informed consent - Need for adequate training and updating in the use of systems for all personnel involved - Not dedicated to the management of emergencies - Periodic verification of the quality of data and diagnostic tools |
| Eichberg et al.  July 2020 | USA | Neurosurgery | Systematic review | Telemedicine in general | - Limited access to technology - Verbal consent - Providers should have a low threshold to convert to a telephone call - Telemedicine neurological exam should be considered a screening exam |
| Ekong et al.  April 2020 | Nigeria | JMIR mHealth and uHealth | Qualitative review | Mobile positioning data | - Balance between deploying technology and maintaining data safety and patient privacy - Informed consent - Protect and safeguard individuals' data by law - A third-party agreement should be formally signed between parties interfacing patient data to protect it |
| Gao et al.  May 2020 | China | Annals of Translational Medicine | Systematic review and meta-analysis | Telemedicine in general | - People were not followed up for outcomes and hotline data were not collected systematically - If the operators do not have enough professional knowledge, they may provide wrong information or provide inappropriate medical advice, leading to a treatment delay or missed diagnoses |
| Jiménez-Rodríguez et al.  July 2020 | Spain | International Journal of  Environmental Research and Public Health | Qualitative review | Video consultations | - Lack of access to the required resources and technological difficulties for both professionals and patients (especially for the elderly) - Some medical procedures are impossible - Lack of technical skills among professionals and patients - Need for training regarding both nontechnical and social-emotional skills - Healthcare professionals were concerned that relationships with their patients may deteriorate - Problems may arise among patients of advanced age, who may have reduced cognitive abilities |
| Kalu et al.  22 August 2020 | UK | Journal of Plastic, Reconstructive, and Aesthetic Surgery | Literature review | Online video consultation platforms and store-and-forward telemedicine | - Time lag and poor audio-visual quality due to insufficient bandwidth - Transparency over the cost, privacy settings, and relative usage of different systems is limited - Patient's identity should first be confirmed - Consent should be gained and recorded - Ensure that internet connection is secure - Reassure patients that their privacy is to be respected - Urgent or serious conditions where physical exam conducted over video consultations may not be appropriate - It is contraindicated to use video consultations when the provider is unsure of the patient's capacity |
| Kaplan et al.  July 2020 | USA | International Journal of Medical Informatics | Literature review | Telemedicine in general | - Technological infrastructure - Access problems (especially the elderly, disabled, or those who have compromised hearing, vision, manual dexterity..) - Confidentiality, privacy, and security require more scrutiny - Informed consent - Ethical concerns - Regulatory issues - Doctor-patient relationship - Patients and clinicians needed to learn how to select and use the technologies - “a whole-system strategy” is suggested to embed telehealth into routine service and other information system functions |
| Khilnani et al.  25 June 2020 | USA | Journal of Information, Communication and Ethics in Society | Case study | Telemedicine in general | - Older adults and those with economic disadvantage are also more likely to experience digital inequality - Long-standing challenges that may impact eHealth adoption, including education, income, broadband access, information-seeking skills and rural residence - eHealth requires a battery of resources and skills on the part of patient and practitioner - Older adult patients as more likely to struggle with skill deficits than younger patients - Digitally disadvantaged are less likely to use eHealth services and thereby bear greater risks during the pandemic to meet ongoing medical care needs during the pandemic |
| Lawrence et al.  8 July 2020 | USA | Journal of General Internal Medicine | Case study | Virtual OSCE* | - Technical challenges can result in significant barriers to communication - Adaptation of traditional components of the medical history and physical exam into the virtual space - Providers may be unable to acquire basic information from remote patients - The diagnostic accuracy of the physical exam maneuvers that are self-executed by patients is not yet known - Residents may not be adequately prepared to provide high-quality care via telemedicine - Needs for both technical proficiency and care delivery quality assurance at both trainee and practitioner levels - Many traditionally employed nonverbal cues may be difficult to deploy and/or interpret, both by patients and providers, in a virtual context - Medical associations recommend at least basic training in technical elements |
| Moss et al.  July 2020 | USA | Journal of Neuro-Ophthalmology | Cross-sectional | Synchronous (video visits) and asynchronous (Store-forward: remote interpretation of tests, second-opinion review, and e-consults) telehealth | - Data quality was selected as the most perceived barrier - Video does not offer much more than phone for ophthalmology - Variable reliability of live video technologies - Video telemedicine visits may take extra time, resulting in decreased clinic volumes - Patient dissatisfaction with billing - Decreased precision and comprehensiveness of examination - More physically draining than face-to-face to maintain engagement with patients - Adoption was greatest in the younger respondents - Provider dissatisfaction - Privacy - Protocols, strategies and scheduling to optimize both efficiency and outcomes and train trainees and providers |
| Mostafa et al.  7 July 2020 | Egypt | Journal of Dermatological Treatment | Cross-sectional | Synchronous (video visits) and store-forward | - Lack of teledermatology consultations in the public hospital bacause of difficult internet connection - No private insurance coverage for teledermatological sevices - Face to face visits are still needed for some conditions like skin cancer check and its surgeries - Showing one part of the body with a skin lesion can be misleading in diagnosis - Simulated teledermatology visits may miss some diagnoses and complications of medications - Legislation is needed |
| Murphy et al.  26 June 2020 | Ireland | Clinical Orthopedics and Related Research | Systematic Review | Virtual clinic model (video and telephone consultations) | - Administrative error regarding the appointment being issued - Adverse outcomes encompass complications, further surgeries, deviations from protocols and re-referrals back to the clinic, inappropriate referrals, mismanagement/misdiagnosis and poorly applied splinting in a specialist hand clinic - Informed consent and agreement with the treatment plan - There must be a way for the patient to contact the service if difficulties arise |
| Ohlstein et al.  2 August 2020 | USA | The Laryngoscope | cross sectional | Video consultations | - An association between age, technical difficulties, and hesitation in the adoption of virtual medicine - Increased complaints of logistic and technical difficulties, especially in older populations - The average age of those declining visits due to technical difficulties was 80 years - Limitation of virtual otoscopic evaluations - Lack of physical exam - Otology patients were less likely to accept a telehealth visit |
| Puro et al.  June 2020 | USA | The Journal of Rural Health | Cross-sectional study | Telehealth and eICU capabilities | - Internet connectivity - Technological restrictions - State reimbursement, regulatory, insurance restrictions play a role in limiting adoptions - Clinician acceptance barriers in general can pose a threat to successful telehealth implementation - Geographic restrictions - The concentrations of rural hospitals possessing these capabilities varied widely by state - Coastal areas lacked to a great extent the capability to provide e-services in rural areas |
| Rametta et al.  June 2020 | USA | Neurology | Qualitative review | Audio-visual telemedicine encounters and scheduled telephone encounters using phones | - The technical quality was impaired, and the most frequent single causes affecting quality were poor audio, poor video, and interruption of the encounter - Patients who lacked access to a smartphone or computer application required to enable telemedicine encounters were scheduled for structured (audio-only) telephone encounters - Access to telemedicine encounters compared to telephone encounters was lower in racial and ethnic minority groups |
| Serper et al.  August 2020 | USA | Hepatology | Case study | Video consultations | - Technical issues were faced due to software upgrades, resulting in one delayed visit on the same day and one visit requiring rescheduling - Payer reimbursement policies are highly variable and most payers do not provide telemedicine parity with in-person visits - Regulatory and financial barriers |
| Sorensen et al.  June 2020 | USA | Annals of Surgery | Cross-sectional study | Video consultation and phone calls | - Preference for in-person versus virtual surgical consultation reflected access to care, with preference for telemedicine decreasing from 72% to 33% when COVID-related social distancing ends - Telemedicine visits are less appropriate for surgical consultation - Concerns about technology related to telemedicine: both functionality and data security - Practical considerations around reimbursement for services and health care utilization will need to be resolved - Physical exam and establishing trust and comfort could best be done in person - Initiating/completing a diagnostic workup would also be better in person - Concern for the depersonalization of care with telemedicine and the ability to establish rapport virtually |
| Tashkandi et al.  June 2020 | Saudi Arabia | Journal of Medical Internet Research | Qualitative cross-sectional study | Virtual visits | - Lack of physical exam - Patients’ awareness and access - IT support and resources were not available - Lack of physical attendance of the patient - Lack of a direct doctor-patient encounter - Medicolegal aspects and privacy - Only 36.0% will continue virtual management after the pandemic |
| Tenforde et al.  May 2020 | USA | PM&R: The Journal of Injury, Function and Rehabilitation | Cross-sectional study | Audiovisual consults | - Limitations in technology and ability to perform a physical examination - Insurance payment models - Access to telehealth technology - Physician knowledge - Malpractice insurance - Concern regarding development of patient rapport - Telehealth visits worked best for follow-up encounters where more limited physical examination was adequate for management recommendations - Barriers in healthcare delivery - Systemic barriers to patients with sensory disability, cognitive deficits, those challenged in using technology or without necessary electronic devices, as well as those who require use of a medical interpreter |
| Triantafillou et al.  July 2020 | USA | Otolaryngology–Head and Neck Surgery | Qualitative cross-sectional | Video-based consultations | - Technical challenges, including issues with connectivity and audio - Various aspects of the doctor-patient relationship were studied, including the video aspect, intimacy of telemedicine, the element of ‘‘human touch,’’ and the physical examination - Anxiety about the logistics of the visit and skepticism regarding telemedicine - Patients preferred in-person visits and did not think that telemedicine visits could replace in-person ones - Remote visits hampered the doctor-patient relationship - The physician could not perform flexible laryngoscopy |
| Wamsley et al.  July 2020 | USA | Aesthetic Surgery Journal | Case Study | Telephone and Video consultations | - Telehealth utilization is lower among 80+ years individuals this may be due to unfamiliarity and lack of comfort with electronic devices and cognitive decline - Reasons for decline of telehealth services included lack of comfort and familiarity with the technology, concerns over privacy and confidentiality, and the preference to schedule an in-person office visit when available - The legal system currently lags behind the available technology - Lack of control over the collection, utilization and sharing of data over the telehealth systems - Informed consent - Malpractice - The practitioner-patient relationship will inevitably be affected - The physical nature of many conventional clinical tests are simply impossible to perform |
| Yoon et al.  June 2020 | USA | International Journal of Spine Surgery, | Qualitative review | Video consultations | - The loss of direct physical examination - The potential for not detecting subtle neurologic deﬁcits - Technical software or hardware difﬁculties - By no means can telemedicine replace all in-person visits - There is no standard method to accurately, reliably, and consistently perform a spine examination through telemedicine - Data privacy is a huge concern - The breach of personal health information can occur despite multiple layers of security - These technical shortfalls may be ameliorated by improving network speed, accessibility, and upgrading software usability |
| Holtz et al. | USA | Telemedicine journal and e-health | Cross-sectional study | Telemedicine in general | - New users of telemedicine perceived more problems hearing the provider through telemedicine more than past users - Difficulty hearing and seeing the health care provider over the computer/mobile system - Privacy - When an unexperienced provider utilizes telemedicine, they might not have the same technical expertise and experience communicating over technology as other telemedicine-only providers - The health care provider spent little time taking medical history - Less communication with the provider - Worries about the accuracy of the information from the telemedicine health care provider - Worries about the continuity of care |
